# The application rate for urology specialty compared with other specialties from 2007 to 2014 in Korea: is it influenced by social interest manifested by internet trends?

**DOI:** 10.1186/s12894-018-0375-y

**Published:** 2018-07-24

**Authors:** Hwa Yeon Sun, Young Myoung Ko, Seung Wook Lee, Bora Lee, Jae Heon Kim

**Affiliations:** 1 Department of Urology, Soonchunhyang University Hospital, Soonchunhyang University College of Medicine, 59, Daesagwan-ro, Yongsan-gu, Seoul, 140-743 South Korea; 2 Department of Industrial and Management Engineering, Pohang University of Science and Technology, Pohang, South Korea; 3 Department of Urology, Hanyang University Guri Hospital, Hanyang University College of Medicine, Seoul, South Korea; 4 Department of Statistics, Graduate School of Chung-Ang University, 84, Heuksukro, Seoul, 156-756 South Korea

**Keywords:** Clinical clerkship, Trends, Employment, Supported, Urology, Education

## Abstract

**Background:**

Reduced clinical exposure to urology at the undergraduate or internship level is the main explanation for the marked decrease in applicants to urology residencies. This manuscript was to access the application rate for urology specialty compared with that of other specialties and to investigate the relationship between the decreasing trend in urology applications and social interest using internet trend tests.

**Methods:**

We reviewed data collected by the Korean Hospital Association from 2007 to 2014. We assessed internet trends using Naver Trend for domestic social interest and Google Trends for international social interest (2007 to 2014). Trend tests and Spearman correlations were used for statistical analyses.

**Results:**

Among the all specialties, the application rates to obstetrics and gynecology, emergency medicine, and occupational medicine are significantly increasing (*p* = 0.015, 0.012, and 0.048, respectively). Application to other specialties is mostly decreasing. The decreasing trend is highest for urology (beta = − 12.21 and *p* < 0.001). The application rate and domestic social interest revealed by Naver trends were significantly correlated (*r* = 0.786 and *p* = 0.021). No correlation was found between Naver trends and Google trends (*r* = − 0.19 and *p* = 0.651).

**Conclusions:**

The rate of application to urology specialty is decreasing the fastest, and this trend is related to domestic social interest. An attempt should be made to increase the number of urologic applicants.

**Electronic supplementary material:**

The online version of this article (10.1186/s12894-018-0375-y) contains supplementary material, which is available to authorized users.

## Background

Urology has been perceived as a competitive specialty for the last decade, but its popularity is decreasing worldwide. A recent report from Canada demonstrated the current status of the reduced popularity of urology using data from the Canadian Residency Matching Service from 2002 to 2011 [1]. Although the number of urology positions has increased, the number of applicants to medical residency specialty who make urology their first choice is decreasing [1, 2].

Considering that the popularity of surgery is also decreasing worldwide, it is not surprising that the popularity of urology is decreasing. However, amongst all surgical specialties, the pattern is most critical in Urology [1, 2].

There is growing concern about this issue because the prevalence of urological diseases, including prostate hyperplasia, overactive bladder, and urological cancers, is increasing [3] and more urologists are necessary from the perspective of public health and the health of individual communities. Reduced clinical exposure to urology at the undergraduate or internship level is the main explanation for the marked decrease in applicants to urology residencies [4].

Although several factors to explain this phenomenon have been noted, including lower early exposure to urology, demand for less difficult specialties associated with greater work-life balance, and a gender shift to female predominance among medical students [1, 2], more explanations are needed to fully account for this sharp decrease, especially in Korea.

The aim of our study is to assess the decrease in the application rate to urology specialty compared with that to other specialties and to investigate the relationship between the decreasing trend in urology applicants and social interest using internet trend testing, which is a validated tool that has been used to explore social interest.

## Methods

### Application rates for medical specialties

We reviewed data collected by the Korean Hospital Association from 2007 to 2014. The application rates to medical specialties, including total applications, internal medicine, pediatrics, neurology, psychiatry, dermatology, rehabilitation, and family medicine were reviewed. The application rates to surgical specialties including overall surgical specialties, general surgery, thoracic surgery, orthopedic surgery, neurosurgery, plastic surgery, obstetrics and gynecology, ophthalmology, otorhinolaryngology, and urology were reviewed. The application rates to other specialties, including the overall rate, anesthesiology, radiology, radiation oncology, laboratory medicine, pathology, emergency medicine, nuclear medicine, occupational medicine, and preventive medicine were reviewed.

### Social interest

Social interest was identified by researching internet trends using Naver Trend and Google Trends. After accessing Naver Trend (http://trend.naver.com/), we searched for each specialty in Korean. The classification was ‘PC version’. The period analyzed was from 2007 to 2014. The trends for the search words were calculated from January to December of each year.

The trend scores were defined as relative indexes. The highest trend score in each year was 100, representing peak search interest and the lowest score was 0, indicating no search entries. The medical specialties investigated were family medicine, neurology, digestive medicine, nephrology, pulmonology, hemato-oncology, rheumatology, endocrinology, infectious disease, cardiology, neurosurgery, orthopedic surgery, thoracic surgery, vascular surgery, plastic surgery, pediatrics I (terminology in Korean before 2007), pediatrics II (terminology in Korean after 2007), obstetrics and gynecology, psychiatry I (terminology in Korean before 2011), psychiatry II (terminology in Korean after 2011), anesthesia, pain medicine, emergency medicine, dermatology, ophthalmology, otorhinolaryngology I (real terminology in Korean), otorhinolaryngology II (similar terminology in Korean), urology, radiology I, radiology II, occupational medicine, preventive medicine, laboratory medicine, and pathology. These were identical as searching terminologies used during trend test.

After accessing Google Trends (http://www.google.com/trends/), we searched for each specialty using the English language and did not limit the geographical region other than to exclude Korea. The category was chosen the ‘All category’ , the classification was selected as ‘web search’ among ‘web search’ , ‘Image search’ , ‘News search’ , ‘Google shopping, and ‘YouTube search’. The method for calculating trend scores was conducted as for Naver trends.

### Statistical analysis

To investigate whether Google and Naver Trend data were related to the application rates in the period from 2007 to 2014, we used a linear regression model; we also used Pearson correlation analysis to analyze the association between Naver trends and the rate of application to urology departments. The trend score is not an absolute value from searched amounts, but rather relative value depending on the flows. Hence, newly calculated calibrated index was adopted using percentage scales: the highest trend score of each period (year) as 100 for retaining objectivity. R (ver3.1.2) was used to analyze and plot data using linear regression and Pearson correlation test. During linear regression test, beta was defined as regression coefficient which represents the estimates of impact. During Pearson correlation analysis, r was defined as correlation coefficient. All tests were two-tailed and statistical significance was set at *p* < 0.05.

## Results

### Application rates to medical residency specialty in Korea from 2007 to 2014

**Fig. 1:**
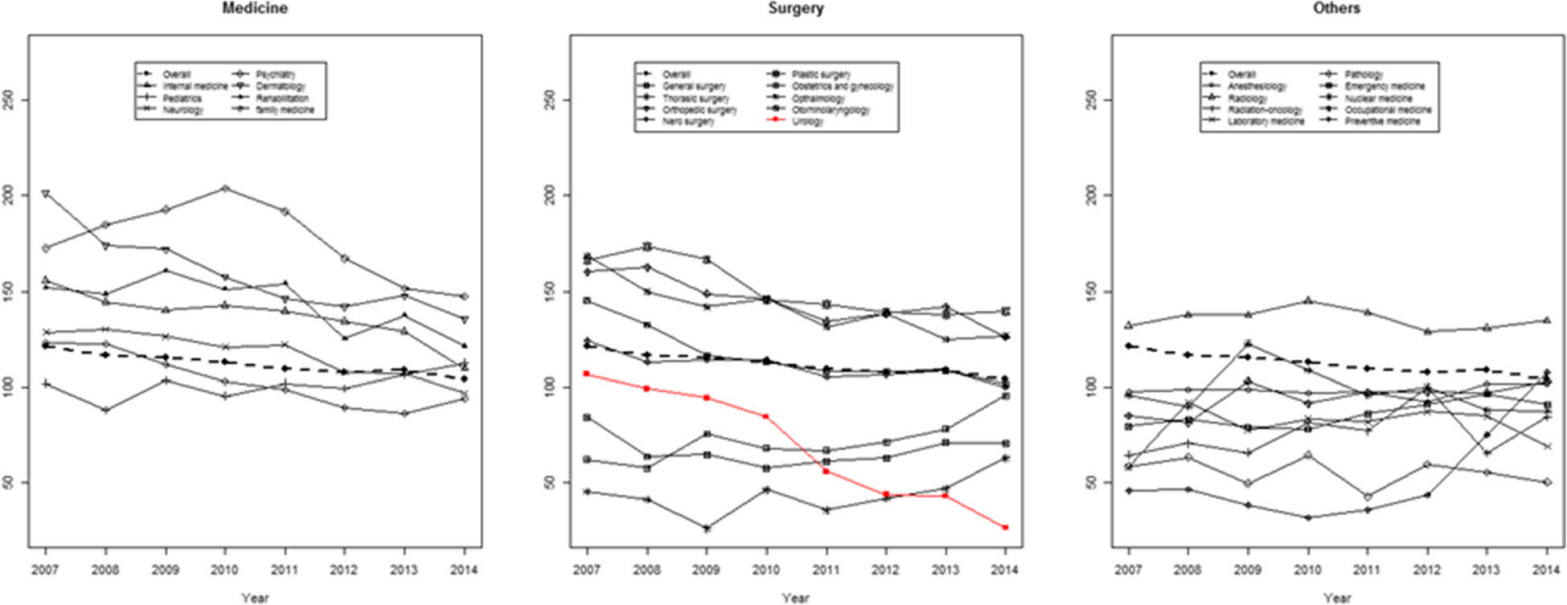
Trends in application rates to residency programs

The application rates to obstetrics and gynecology, emergency medicine, and occupational medicine programs significantly increased over the study period (*p* = 0.015, 0.012, and 0.048, respectively) (Fig. 1). The application rates to other specialties mostly decreased (Fig. 1). The extent of the decrease is greatest for urology (beta = − 12.21 and *p* < 0.001) (Additional file 1: Table S1).

### Trend for each specialty as assessed using Naver trend

**Fig. 2:**
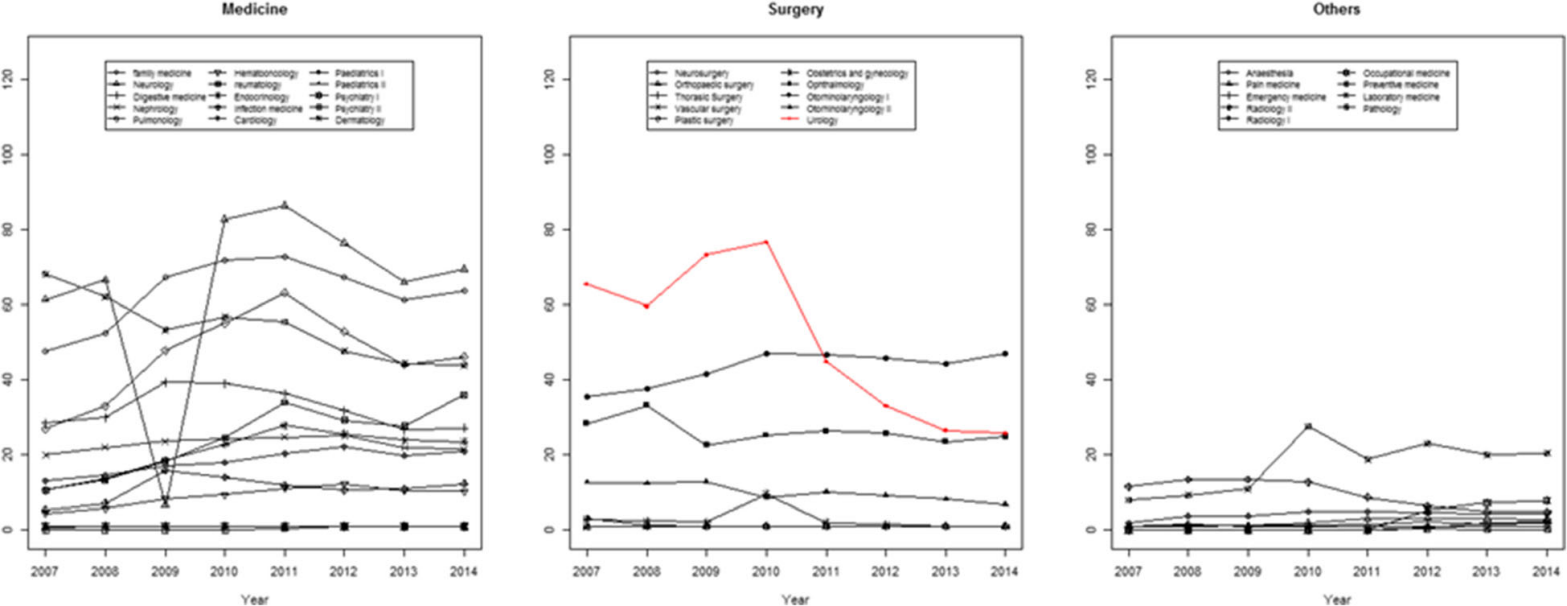
Trends in domestic social interest for each specialties assessed using Naver trend

Naver Trend was used to investigate the domestic social interest for each specialty within Korea (Fig. 2). Among the medical specialties, significantly increasing social interest was noted in hemato-oncology, rheumatology, endocrinology, cardiology, and psychiatry, whereas only dermatology showed significantly decreasing social interest (Additional file 2: Table S2). Among the surgical specialties, ophthalmology and urology showed significantly decreasing social interest. For urology, the beta value was highest, − 7.10 (*p* = 0.01) (Additional file 2: Table S2). For other specialties, significant increasing trends were noted in radiology, occupational medicine, and preventive medicine (Additional file 2: Table S2).

### Trend for each specialty as assessed using Google trends

**Fig. 3:**
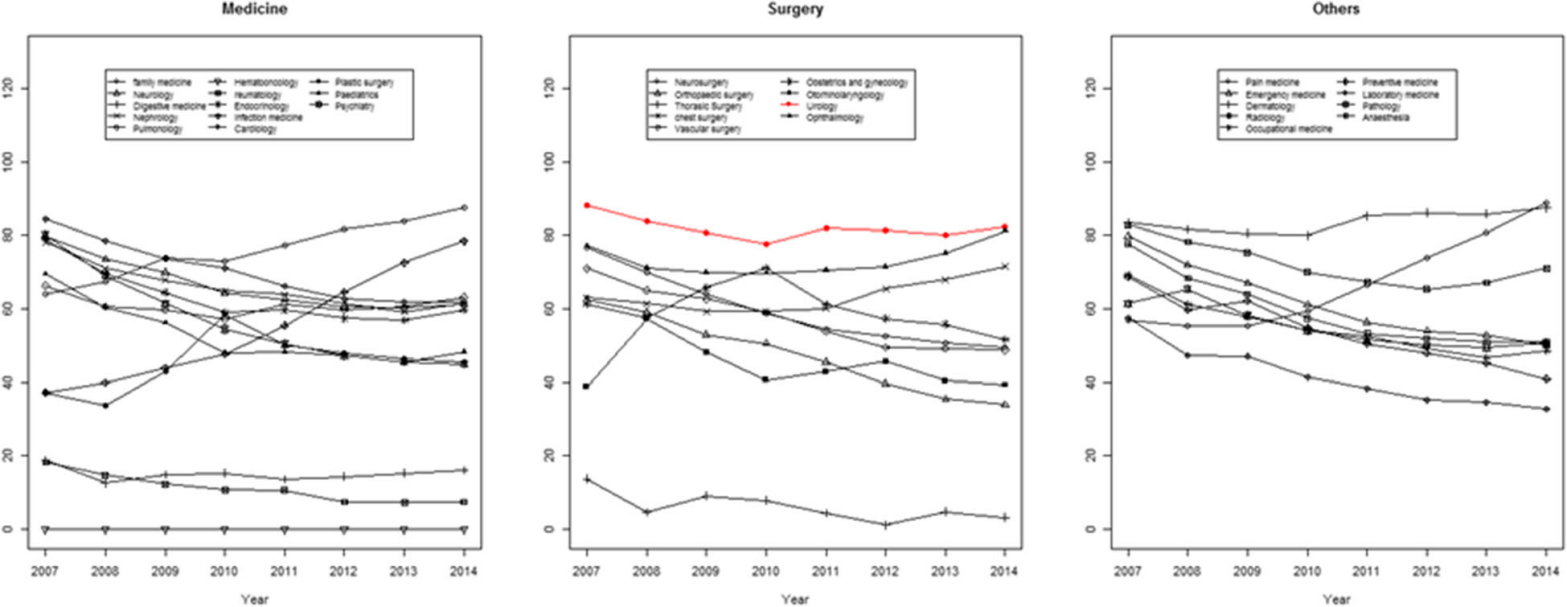
Trends in international social interest for each specialties assessed using Google trend

Google Trend was used to investigate the international social interest for each specialty (Fig. 3). For medical specialties, a significant increasing trend was noted in family medicine, infectious diseases, and dermatology (Additional file 3: Table S3). A significant decreasing trend was noted in neurology, nephrology, rheumatology, endocrinology, cardiology, and pediatrics (Additional file 3: Table S3). Among the surgical specialties, a significant decreasing trend was noted in neurosurgery, orthopedic surgery, thoracic surgery, and vascular surgery (Additional file 3: Table S3). Only chest surgery showed a significant increasing trend. For other specialties, a significant increasing trend was noted in pain medicine and dermatology, whereas a significant decreasing trend was noted in anesthesia, radiology, preventive medicine, occupational medicine, laboratory medicine, and pathology (Additional file 3: Table S3). For urology, no significant trend was noted.

### Correlation between application rate and social interest

**Fig. 4:**
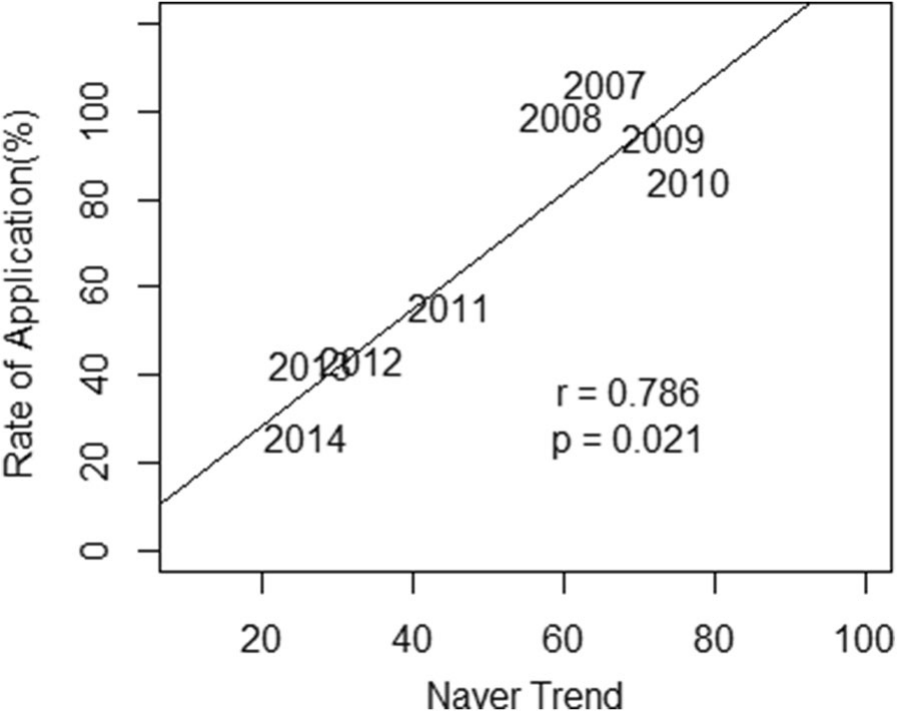
Correlations between the application rate for resident training and social interest

For urology, the application rate and domestic social interest as revealed by Naver Trend were significantly correlated (*r* = 0.786 and *p* = 0.021) (Fig. 4). Out of all specialties, only pain medicine was similar in Naver and Google trends, revealing a significant increase in domestic and worldwide social interest.

## Discussion

The hypothesis of this study was that application rate could be associated with social interest, which was confirmed by our analysis. Basic assumption of this hypothesis was that general interest regarding urologic specific disease is decreasing. To prove this basic assumption, we also investigated the social trend using Naver and Google trend about urologic cancers, which showed the decreasing trend as basic assumption (Additional file 4: Figure S1).

Traditionally, urology has been perceived as a competitive and popular surgical specialty. The main attraction of urology for prospective trainees was that urology consists of a mixture of medical and surgical specialties and those trainees can begin working in a clinical setting earlier than they would in other disciplines [5]. However, today, there is a decrease in the number of applicants to surgical specialty specialty in general [6–8], and the competitiveness and popularity of urology are decreasing [1]; this phenomenon is happening extremely quickly in Korea.

In recent years, surgical specialties have become less competitive and less popular among medical students [6–8]. Lind et al. reported that the number of medical students that chose to enter surgical fields decreased from 22% in 1982 to 15% in 2002 [9]. This large shift is attributable to the increasing interest in lifestyle factors [10]. More and more students are concentrating on their lives outside of work [11], so they enter fields that provide more flexible work hours and allow them to enjoy their private lives [10].

Before the 2000s, although there was a decreasing trend in surgery as a career choice, surgical specialties were still competitive and popular because they were associated with good career opportunities and prestige [12]. However, in the early 2000s, lifestyle factors became more important and negatively affected the number of individuals choosing surgery as their careers [10, 12]; other factors that influence this decreasing trend include long work hours during residency and the quality of the patient/physician relationship [12].

Nowadays, because of the effect of the negative factors mentioned above, the rate of applications to surgical fields is decreasing. Applications to urology specialty are decreasing at the fastest rate. Like in other countries, the application rate to urology specialty in Korea is decreasing with the highest slope (Fig. 1). The application rate to urology specialty in Korea is the lowest out of surgical specialties and all specialties.

One important factor in this change is the increasing percentage of female medical students. In Canada, among all medical students enrolled in 2010, more than half were female [1]. The percentage of female students is increasing; it went from 50.1% in 2002 to 57.1% in 2011 [1]. A review of application data from 2002 to 2011 revealed that, although female applicants to surgical specialties almost doubled (21 to 41%), there was no change in the percentage of medical students going into urology [1].

Besides the gender shift, there are several other factors that help to explain the rapid decrease in urology applicants. First, although there are benefits of urology, including earlier experience in the clinical field and a diversity of procedures, Kerfoot et al. found that the pathology of disease aspect of the specialty could be perceived as having a repetitive nature and that urology residencies are demanding of students’ time [5, 13]. Second, during medical school, there is too little exposure to urology. Malde et al. reported that 26.6% of medical students in the UK had no urology experience throughout their clinical clerkship period. Only 30.5% of medical students were exposed to urology during their clerkships; also, the clerkship period for urology was limited to 1 week [14]. The same phenomenon was noted in the US: medical students in the US can complete their clinical clerkships without being exposed to urology. Kutikov et al. reported that length of clinical experience in urology strongly correlated with the number of students choosing urology for their careers [15].

However, even the reasons mentioned above, including gender shift, repetitive nature of some of the work, and scanty exposure to urology during the medical clerkship period, do not fully explain the extraordinary rate at which the number of urology applicants is decreasing. To identify reasons having to do with social interest, we investigated domestic and international internet trends. Korea has a high population density, and social interest may be more uniform than it is in other countries [16]. Internet trend testing is a validated method used to investigate social interest. For example, it has been used to assess the interest in bariatric surgery [17]. Internet search volume is based on social interest and several analyses have revealed that search volume trends reflect real-world events. Therefore, Google Trend is a useful tool to investigate the social interest in various clinical fields [18–21]. Moreover, Naver is more popular searching engine in Korea and Naver trend is a good tool to reflect the general social interest in Korea [22]. One more factor that may impact the near future is the system of limitation of resident working time. Limitation of resident working hours is still and will continue to be a hotly controversial topic. One hopeful study showed that increasing the time medical students are exposed to urology and decreasing the size of academic units, which makes the units more comfortable for the students, can increase students’ interest level and, therefore, the number of applicants [23].

### Limitations

Although our study represents a novel pilot research, it has several limitations. First, the medical education system is not uniform and each country has its own medical curriculum. However, even though the time at which students must choose their medical specialty differs between countries (in some countries it is during medical school, whereas in others it is during the internship period), students everywhere still must make that decision by themselves. Second, competition rate and application rate are different concepts. Some specialties have become more competitive because they reduced the quota for the number of applicants. However, the absolute application rate is far more important than the competition rate. Third, those data used in this study is not an individual unit data, rather a panel data based by year, which yield limitation during expansion of analysis. Lastly, this study did not consider specific social factors including the state of the job market or financial compensation. However, several studies have already documented such factors, including lifestyle factors, career ambitions, family status, finances, travel conditions, and research [2, 10, 12, 24]. Personal factors including life style or working hours are becoming an important deterrent factor to choose surgery including urology as a career choice [11, 12, 25].

## Conclusions

In summary, the application rate for urology is decreasing at the fastest rate, and this trend is related to a decrease in domestic social interest. Various attempts must be made to determine the total number of urologic specialties using scientific method are needed.

## Additional files

Additional file 1: **Table S1.** Application rate to medical residencies in Korea (Trend test from 2007 to 2014). (DOCX 16 kb)

Additional file 2: **Table S2.** Social interest as assessed by Naver trends from 2007 to 2014. (DOCX 17 kb)

Additional file 3: **Table S3.** Social interest as assessed by Google trends from 2007 to 2014. (DOCX 18 kb)

Additional file 4: **Figure S1.** Trends in social interest of bladder cancer and prostate cancer for each specialties assessed using Naver and Google trend. (TIF 158 kb)
