# Supplementary material for: The application rate for urology specialty compared with other specialties from 2007 to 2014 in Korea: is it influenced by social interest manifested by internet trends?
Source: BMC Urol. 2018 Jul 24;18:65. doi: 10.1186/s12894-018-0375-y (PMC6057043; doi:10.1186/s12894-018-0375-y)
Supplement: Supplementary file 1 — Table S1. Application rate to medical residencies in Korea (Trend test from 2007 to 2014). (DOCX 16 kb) [file 12894_2018_375_MOESM1_ESM.docx]

**Additional file Table S1. Application rate to medical residencies in Korea (Trend test from 2007 to 2014)**

|  | beta | p-value |
| --- | --- | --- |
| overall | -2.20 | <0.001 |
| Medicine |  |  |
| Dermatology | -8.28 | <0.001 |
| Family medicine | -5.45 | <0.001 |
| Internal medicine | -4.98 | 0.002 |
| Neurology | -4.68 | <0.001 |
| Pediatrics | 1.92 | 0.085 |
| Psychiatry | -5.16 | 0.01 |
| Rehabilitation | -4.45 | 0.028 |
| Surgery |  |  |
| General surgery | -0.71 | 0.621 |
| Nero surgery | -2.67 | 0.004 |
| Obstetrics and gynecology | 3.86 | 0.015 |
| Ophthalmology | -5.28 | 0.002 |
| Orthopedic surgery | -4.60 | 0.002 |
| Otorhinolaryngology | -5.41 | 0.002 |
| Plastic surgery | -5.37 | 0.003 |
| Thoracic surgery | 2.23 | 0.184 |
| Urology | -12.21 | <0.001 |
| Others |  |  |
| Anesthesiology | 0.33 | 0.531 |
| Emergency medicine | 2.27 | 0.012 |
|  |  |  |
| Laboratory medicine | 0.80 | 0.673 |
| Nuclear medicine | -1.77 | 0.381 |
| Occupational medicine | 2.31 | 0.048 |
| Pathology | -1.06 | 0.399 |
| Preventive medicine | 7.12 | 0.066 |
| Radiation-oncology | 2.56 | 0.197 |
| Radiology | -0.59 | 0.508 |
