# Supplementary material for: The application rate for urology specialty compared with other specialties from 2007 to 2014 in Korea: is it influenced by social interest manifested by internet trends?
Source: BMC Urol. 2018 Jul 24;18:65. doi: 10.1186/s12894-018-0375-y (PMC6057043; doi:10.1186/s12894-018-0375-y)
Supplement: Supplementary file 2 — Table S2. Social interest as assessed by Naver trends from 2007 to 2014. (DOCX 17 kb) [file 12894_2018_375_MOESM2_ESM.docx]

**Additional file 2: Table S2. Social interest as assessed by Naver trends from 2007 to 2014**

|  | beta | p-value |
| --- | --- | --- |
| Medicine |  |  |
| Cardiology | 1.18 | 0.002 |
| Digestive medicine | -0.61 | 0.493 |
| Dermatology | -3.28 | <0.001 |
| Endocrinology | 2.00 | 0.014 |
| Family medicine | 1.85 | 0.201 |
| Hemato-oncology | 0.93 | 0.009 |
| Infection medicine | 0.59 | 0.296 |
| Nephrology | 0.18 | 0.575 |
| Neurology | 3.17 | 0.452 |
| Pediatrics I | 0.00 | 0.134 |
| Pediatrics II | 0.03 | 0.123 |
| Psychiatry I | 0.00 | 0.134 |
| Psychiatry II | 0.18 | 0.002 |
| Pulmonology | 2.54 | 0.177 |
| Rheumatology | 3.49 | 0.002 |
| Surgery |  |  |
| Neurosurgery | 0.00 | 0.134 |
| Obstetrics and gynecology | -0.35 | 0.454 |
| Ophthalmology | -0.74 | 0.16 |
| Orthopedic surgery | 0.00 | 0.134 |
| Otorhinolaryngology I | 1.51 | 0.136 |
| Otorhinolaryngology II | -0.85 | 0.002 |
| Plastic surgery | -0.19 | 0.092 |
| Thoracic Surgery | 0.00 | 0.134 |
| Urology | -7.10 | 0.01 |
| Vascular surgery | 0.01 | 0.134 |
| Others |  |  |
| Anesthesia | -0.03 | 0.186 |
| Emergency medicine | 0.11 | 0.034 |
| Laboratory medicine | 2.02 | 0.055 |
| Occupational medicine | 1.26 | 0.005 |
| Pain medicine | 0.37 | 0.002 |
| Pathology | 0.02 | 0.189 |
| Preventive medicine | 0.31 | 0.006 |
| Radiology I | 0.26 | 0.082 |
| Radiology II | -1.36 | 0.003 |
