# Supplementary material for: The application rate for urology specialty compared with other specialties from 2007 to 2014 in Korea: is it influenced by social interest manifested by internet trends?
Source: BMC Urol. 2018 Jul 24;18:65. doi: 10.1186/s12894-018-0375-y (PMC6057043; doi:10.1186/s12894-018-0375-y)
Supplement: Supplementary file 3 — Table S3. Social interest as assessed by Google trends from 2007 to 2014. (DOCX 18 kb) [file 12894_2018_375_MOESM3_ESM.docx]

**Additional file 3: Table S3. Social interest as assessed by Google trends from 2007 to 2014**

|  | beta | p-value |
| --- | --- | --- |
| Medicine |  |  |
| Cardiology | -3.30 | <0.001 |
| Dermatology | 0.85 | 0.035 |
| Digestive medicine | -0.11 | 0.732 |
| Endocrinology | -2.59 | 0.01 |
| Family medicine | 3.26 | <0.001 |
| Infection medicine | 6.21 | <0.001 |
| Nephrology | -2.32 | 0.001 |
| Neurology | -2.68 | 0.001 |
| Pediatrics | -2.98 | 0.005 |
| Psychiatry | -4.89 | <0.001 |
| Pulmonology | -0.20 | 0.669 |
| Rheumatology | -1.52 | <0.001 |
| Surgery |  |  |
| Chest surgery | 1.33 | 0.04 |
| Neurosurgery | -3.88 | <0.001 |
| Obstetrics and gynecology | -1.33 | 0.172 |
| Ophthalmology | 0.64 | 0.356 |
| Orthopedic surgery | -4.32 | <0.001 |
| Otorhinolaryngology | -1.03 | 0.32 |
| Plastic surgery | 1.53 | 0.22 |
| Thoracic Surgery | -1.19 | 0.037 |
| Urology | -0.62 | 0.206 |
| Vascular surgery | -3.33 | <0.001 |
| Others |  |  |
| Anesthesia | -3.75 | <0.001 |
| Emergency medicine | -4.14 | <0.001 |
| Laboratory medicine | -3.27 | <0.001 |
| Occupational medicine | -2.93 | <0.001 |
| Pain medicine | 4.93 | <0.001 |
| Pathology | -2.16 | 0.003 |
| Preventive medicine | -3.73 | <0.001 |
| Radiology | -2.03 | 0.014 |
