# Supplementary figures and images for: The application rate for urology specialty compared with other specialties from 2007 to 2014 in Korea: is it influenced by social interest manifested by internet trends?
Source: BMC Urol. 2018 Jul 24;18:65. doi: 10.1186/s12894-018-0375-y (PMC6057043; doi:10.1186/s12894-018-0375-y)

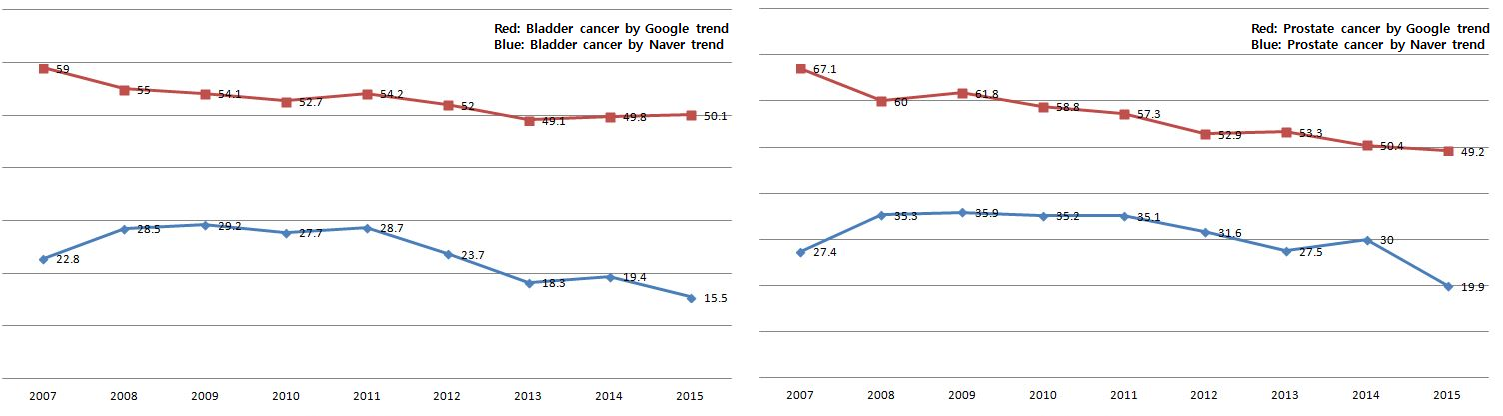

Supplement: Supplementary file 4 — Figure S1. Trends in social interest of bladder cancer and prostate cancer for each specialties assessed using Naver and Google trend. (TIF 158 kb) [file 12894_2018_375_MOESM4_ESM.tif]
